# Determinants of 30-day Morbidity in Adult Cranioplasty: An ACS-NSQIP Analysis of 697 Cases

**DOI:** 10.1097/GOX.0000000000002562

**Published:** 2019-12-11

**Authors:** Rachel E. Armstrong, Marco F. Ellis

**Affiliations:** From the * Feinberg School of Medicine, Northwestern University, Chicago, Ill.; † Division of Plastic Surgery, Feinberg School of Medicine, Northwestern University, Chicago, Ill.

## Abstract

Cranioplasty is performed to restore the function and anatomy of the skull. Many techniques are used, including replacement of the bone flap and reconstruction with autologous or synthetic materials. This study describes the complication profile of adult cranioplasty using a prospective national sample and identifies risk factors for 30-day morbidity.

**Methods:**

The American College of Surgeon’s National Surgery Quality Improvement Project database for 2015–2016 was utilized. Cases were identified by current procedural terminology code, size, and type (autologous/alloplastic). χ_2_, Fisher exact, and ANOVA tests compared demographic differences. Univariate and multivariate logistic regressions were performed to identify risk factors for 30-day morbidity and mortality.

**Results:**

Six hundred ninety-seven cranioplasty cases were identified. Two cases used 2 types of cranioplasties and were counted in both groups. Five hundred forty-three cranioplasties were alloplastic, 57 were autologous, and 99 were classified as “Other.” Age, race, diabetes, ventilator dependency, congestive heart failure, hypertension, wound infection, sepsis, and bleeding disorders were identified on univariate analysis to increase complication risk. Multivariate analysis identified age of the patient, systemic sepsis, and bleeding disorders as significant risk factors for complications. There was no difference in complications between cranioplasty types. Overall and medical complications were greater in cranioplasties >5 cm (*P* < 0.001).

**Conclusions:**

Cranioplasty is a morbid procedure, with a complication rate of 27.4% and a mortality rate of 3.0% in this national sample. Factors such as age, sepsis, bleeding disorders, and size increase risk. Identification and modification of risk factors may guide operative timing and influence informed consent.

## Abstract 2

Supplemental Digital Content is available in the text.

## INTRODUCTION

### Rationale and Background Information

Cranioplasty is performed to replace missing or damaged cranium following craniectomies and craniotomies. Studies have demonstrated clear benefits of this procedure, including appearance restoration, cognitive function improvement, increased cerebral blood flow, and increased patient satisfaction and quality of life.^1–3^ Cranioplasty has an extensive history of use and can be performed with autologous bone or a variety of alloplastic implants.^4,5^

While there have been single institution^6–8^ and nationwide^9^ studies demonstrating that cranioplasty conveys significant risk, there have been no studies to date examining morbidity and mortality of the cranioplasty procedure using a nationally validated, peer-controlled database.^10^ Such a study would provide patients and providers with an understanding of risks for postoperative complication. This would allow for more informed decision-making before surgery and help to guide the timing of cranioplasty procedures.

The American College of Surgeons National Surgical Quality Improvement Program is a national surgical database that contains demographic, comorbidity, operative, and complication data from hundreds of participating hospitals across the United States.^11^ The magnitude and reliability of this database made it an ideal tool with which to examine the morbidity and mortality of cranioplasty.

### Study Goals and Objectives

This study aims to utilize the NSQIP database to assess (1) the risk factors associated with all-cause complication following cranioplasty, (2) the effect of size of cranioplasty implant on complication rates, and (3) the effect of cranioplasty type on complication rates.

## METHODS

### Study Design

The NSQIP database for the years 2015–2016 was surveyed for patients who had undergone cranioplasty. Cases were identified based on current procedural terminology (CPT) codes. Cases were subgrouped according to whether the patients received an alloplastic (CPT 62140 and 62141), autologous (CPT 62146 and 62147), or other type of cranioplasty—including “replacement of bone flap or prosthetic plate of skull” and “cranioplasty for skull defect with reparative brain surgery” (CPT 62143 and 62145). In the autologous and alloplastic groups, cases were categorized based on whether the implant was <5 cm (CPT 62140 and 62146) or >5 cm (CPT 62141 and 62147).

### Variables Studied

Demographic, comorbidity, and operative characteristic data were collected including age, gender, race, diabetes, smoking status, dyspnea, ventilator dependency, COPD, ascites, congestive heart failure within the past 30 days, hypertension, acute renal failure, dialysis status, disseminated cancer, steroid use for chronic condition, >10% loss in body weight in the last 6 months, systemic sepsis, bleeding disorders, pre-op transfusion of >1 units of RBC, inpatient status, wound class, ASA class, total wRVU, and total operative time. BMI was calculated from height and weight data.

Surgical and medical complications were tabulated. Surgical complications included superficial infection, wound infection, organ space SSI, and wound disruption. Medical complications included pneumonia, reintubation, pulmonary embolism, failure to wean from respirator, progressive renal insufficiency, acute renal failure, UTI occurrence, CVA/stroke with neurological deficit, cardiac arrest requiring CPR, myocardial infarction, bleeding transfusions, DVT/thrombophlebitis, sepsis, and septic shock. Overall complications rates included all medical complications and surgical complications, as well as death within 30 days of surgery, any readmission, and unplanned reoperation.

### Missing Data

Patients with missing data were excluded from the analyses that corresponded with their missing data. Thirty-two patients lacked weight and/or height data, and therefore their BMI could not be calculated. One patient’s age was coded as 90+, and thus this patient was excluded from the age data. This patient was also excluded from the multivariate analysis. Sample sizes are listed next to the age and BMI data, the 2 variables in which there was missing data.

### Statistical Analysis

Statistical analyses were conducted using IBM SPSS Statistics 25. Demographic analysis was ascertained utilizing descriptive statistics including the Fisher exact test and χ^2^ test for categorical data and ANOVA for numerical data. The univariate analysis was also performed with the χ^2^ test, the Fisher exact test, and ANOVA. The multivariate analysis was performed using binary logistic regression including the data in the univariate analysis found to be significant or approaching significance. A *P*-value < 0.05 was deemed significant, and *P*-values < 0.10 were deemed as approaching significance.

## RESULTS

From the study population (2015–2016 NSQIP data), 697 patients were included. Of the 697 patients, 1 patient received both an alloplastic and autologous cranioplasty, and 1 patient received both an alloplastic and “other” cranioplasty. These 2 cases were included in both of the corresponding groups. In total, 543 cranioplasties were alloplastic, 57 were autologous, and 99 were classified as “other.”

Demographic, comorbidity, and operative characteristic data between the cranioplasty types is described in Table 1. Demographic, comorbidity, and operative characteristics were compared between the autologous, alloplastic, and “other” subgroups (Table 1). Age (*P* = 0.028), race (*P* <0.001), disseminated cancer (*P* = 0.023), systemic sepsis (*P* = 0.002), ASA class (*P* = 0.002), total wRVU (*P* <0.001), and total operative time (*P* = 0.025) were found to significantly differ between the types of cranioplasty procedures.

**Table 1.** Demographic Data, Comorbidities, and Operative Data Were Compared in Patients with Alloplastic Versus Autologous Versus Other Cranioplasty Types

	Cranioplasty Type						*P*
	Alloplastic		Autologous		Other		
	n = 543	%	n = 57	%	n = 99	%	
Demographics							
Age (n = 698)	55.34 ± 15.86 (n = 542)		49.84 ± 15.68 (n = 57)		56.36 ±1 4.195 (n = 99)		0.028*
BMI (n = 667)	28.93 ± 7.20 (n = 530)		31.21 ± 7.85 (n = 52)		29.445 ± 6.54 (n = 85)		0.087
Gender							0.15
Male	231	42.50	20	35.10	50	50.50	
Female	312	57.50	37	64.90	49	49.50	
Race							**<0.001**‡
American Indian or Alaska	3	0.60	1	1.8	0	0.0	
Native							
Asian	19	3.50	2	3.5	2	2.0	
Black or African American	52	9.60	7	12.3	4	4.0	
Native Hawaiian or Pacific	1	0.20	0	0.0	0	0.0	
Islander							
Unknown/not reported	69	12.70	15	26.3	43	43.4	
White	399	73.50	32	56.1	50	50.5	
Comorbidities							
Diabetes							0.32
Insulin	24	4.4	1	1.8	5	5.1	
Non-insulin	44	8.1	5	8.8	14	14.1	
Current smoker	112	20.6	14	24.6	18	18.2	0.62
Dyspnea							0.81
At rest	3	0.6	0	0.0	1	1.0	
Moderate exertion	19	3.5	2	3.5	2	2.0	
Ventilator dependent	6	1.1	0	0.0	2	2.0	0.53
COPD	21	3.9	0	0.0	4	4.0	0.43
Ascites	0	0.0	0	0.0	0	0.0	
CHF <30 d	2	0.4	0	0.0	1	1.0	0.53
Hypertension	199	36.6	20	35.1	41	41.4	0.62
Acute renal failure	0	0.0	0	0.0	1	1.0	0.22
Currently on dialysis	2	0.4	0	0.0	0	0.0	1.00
Disseminated cancer	89	16.4	3	5.3	21	21.2	0.023*
Open wound/wound infection	17	3.1	0	0.0	4	4.0	0.34
Steroid use for chronic condition	51	9.4	5	8.8	12	12.1	0.67
>10% loss body weight in last 6 mo	9	1.2	0	0.0	4	4.0	0.18
Systemic sepsis (any)							**0.002†**
Sepsis	0	0.00	1	1.80	2	2.0	
Septic shock	0	0.00	0	0.00	1	1.0	
SIRS	27	5.00	0	0.00	2	2.0	
Bleeding disorders	16	2.90	3	5.30	3	3.0	0.62
Pre-op transfusion >1 units RBC	2	0.40	0	0.00	1	1.0	0.53
Inpatient status	527	97.10	55	96.50	98	99.0	0.58
Operative Characteristics							
Wound class							0.24
1. Clean	488	89.90	47	82.50	90	90.9	
2. Clean-contaminated	32	5.90	5	8.80	6	6.1	
3. Contaminated	12	2.20	3	5.30	0	0.0	
4. Infected	11	2.00	2	3.50	3	3.0	
ASA class							**0.002†**
Class 1	16	2.90	3	5.30	0	0	
Class 2	151	27.30	22	38.60	20	20.20	
Class 3	308	56.70	27	47.40	53	53.50	
Class 4	62	11.40	4	7.00	25	25.30	
Class 5	1	0.20	0	0	1	1.00	
None assigned	5	0.90	1	1.80	0	0	
Total wRVU	37.8409±12.17796		33.5663±8.93116		32.5189±8.19076		**<0.001‡**
Total operative time	262.8±163.703		290±183.897		222.37±141.969		0.025*

Significant findings are in bold font.

* *P* value < 0.05.

† *P* value < 0.01.

‡ *P* value < 0.001.

CHF, congestive heart failure.

Complication frequencies are described in Table 2. Twenty-one (3.0%) patients died within 30 days of surgery. Many patients had >1 complication, and a total of 191 patients (27.4%) from the sample experienced 1 or more complications.

**Table 2.** Complication Numbers and Percentages Are Listed for the Entire Sample Size of Patients Who Underwent Cranioplasty

Outcome	Count	Percentage
Surgical complications		
Occurrences superficial infection (SUPINFEC)	7	1.0
Occurrences deep incisional SSI (WNDINF)	4	0.6
Occurrences organ space SSI (ORGSPSSI)	11	1.6
Occurrences wound disruption (DEHIS)	6	0.9
Medical complications		
Occurrences pneumonia (OUPNEUMO)	17	2.4
Occurrences reintubation (REINTUB)	15	2.2
Occurrence pulmonary embolism (PULEMBOL)	4	0.6
Occurrence failure to wean from respirator (FAILWEAN)	28	4.0
Occurrences progressive renal insufficiency (RENAINSF)	4	0.6
Occurrences acute renal failure (OPRENAFL)	2	0.3
Occurrences UTI (URNINFEC)	14	2.0
CVA/stroke with neurological deficit (CNSCVA)	15	2.2
Occurrences cardiac arrest requiring CPR (CDARREST)	3	0.4
Occurrences myocardial infarction (CDMI)	0	0.0
Occurrences bleeding transfusions (OTHBLEED)	58	8.3
Occurrences DVT/thrombophlebitis (OTHDVT)	17	2.4
Occurrences sepsis (OTHSYSEP)	17	2.4
Occurrences septic shock (OTHSESHOCK)	1	0.1
Death, readmission, and reoperation		
Death	21	3.0
Readmission	70	10.0
Return to OR	56	8.0
Total		
No. patients with 1+ complications	191	27.4

In the univariate analysis (Table 3), age (*P* < 0.001), race (*P* = 0.030), diabetes (*P* = 0.046), ventilator dependency (*P* = 0.039), congestive heart failure (*P* = 0.020), hypertension (*P* = 0.001), open wound/wound infection (*P* = 0.013), systemic sepsis (*P* = 0.005), and bleeding disorders (*P* = 0.007) were found to be significantly correlated with any complication outcome. Dialysis status (*P* = 0.075) and >10% loss of body weight (*P* = 0.053) approached significance, and were thus included in the multivariate analysis.

**Table 3.** In the Univariate Analysis, Demographics, Comorbidities, and Operative Data Were Compared in Patients Who Developed Complications within 30 Days Versus Patients Who Did Not

	Complications	No Complications	P
Demographics			
Age (n = 696)	58.47 ± 15.84	53.77 ± 15.42	<0.001‡
BMI (n = 665)	29.66 ± 7.70	29.01 ± 6.97	0.30
	Complication occurrencesTotal n = 697	Percentage	
Gender			0.67
Male (n = 300)	85	28.30	
Female (n = 397)	106	26.70	
Race			0.030*
American Indian or Alaska Native (n = 4)	0	0	
Asian (n =23)	6	26.10	
Black or African American (n = 63)	26	41.30	
Native Hawaiian or Pacific Islander (n = 1)	1	100	
White (n = 480)	131	27.30	
Unknown/Not Reported (n = 126)	27	21.40	
Comorbidities			
Diabetes			0.046*
Diabetic with insulin (n = 30)	14	46.70	
Diabetic with non-insulin agents (n = 63)	19	30.20	
Not diabetic (n = 604)	158	26.20	
Current smoker			1.00
Smoker (n = 144)	39	27.10	
Nonsmoker (n = 553)	152	27.50	
Dyspnea			0.18
Dyspnea at rest (n = 4)	1	25	
Dyspnea with moderate exertion (n = 23)	10	43.50	
No Dyspnea (n = 670)	180	26.90	
Ventilator dependent			0.039*
Dependent (n = 8)	5	62.50	
Not dependent (n = 689)	186	27.00	
COPD			0.17
History of severe COPD (n = 25)	10	40	
No COPD history (n = 672)	181	26.90	
CHF <30 d			0.020*
Congestive heart failure (n = 3)	3	100	
No heart failure (n = 694)	188	27.10	
Hypertension			0.001†
Hypertension requiring medication (n = 260)	91	35.00	
No hypertension (n = 437)	100	22.90	
Acute renal failure			0.27
Yes (n = 1)	1	100	
No (n = 696)	190	27.30	
Currently on dialysis			0.075
Yes (n = 2)	2	100	
No (n = 695)	189	27.20	
Disseminated cancer			0.73
Yes (n = 113)	29	25.70	
No (n = 584)	162	27.70	
Open wound/wound infection			0.013*
Yes (n = 21)	11	52.40	
No (n = 676)	180	26.60	
Steroid use for chronic condition			0.67
Steroid use for chronic condition (n = 67)	20	29.90	
No steroid use (n = 630)	171	27.10	
>10% loss body weight in last 6 months			0.053
Yes (n = 13)	7	53.80	
No (n = 684)	184	26.90	
Systemic sepsis (any)			0.005†
Sepsis (n = 3)	2	66.70	
Septic Shock (n = 1)	1	100	
SIRS (n = 29)	14	48.30	
None (n = 664)	174	26.20	
Inpatient status			0.31
Inpatient (n = 678)	188	27.70	
Outpatient (n = 19)	3	15.80	
Bleeding disorders			0.007†
Yes (n = 22)	12	54.50	
No (n = 675)	179	26.50	
Transfusion of ≥1 units RBC 72 hours before surgery			1.00
Yes (n = 3)	1	33.30	
No (n = 694)	190	27.40	

Significant findings are in bold font.

CHF, congestive heart failure.

In the multivariate analysis (Table 4), age (*P* = 0.029), systemic sepsis (*P* = 0.015), open wound/wound infection (*P* = 0.046), and bleeding disorders (*P* = 0.045) were found to be significantly correlated with any complication outcome.

**Table 4.** In the Multivariate Analysis, Factors Found in the Univariate Analysis to Be Significant (*P* < 0.05) or Approaching Significance (*P* < 0.10) Were Evaluated for Significance in a Binary Logistic Regression

	Odds Ratio	95% Confidence Interval		Sig.
		Lower	Upper	
Age of patient with patients over 89 coded as 90+	1.014	1.001	1.027	0.029*
White				0.426
American Indian or Alaska Native	0.000	0.000		0.999
Asian	1.201	0.456	3.163	0.710
Black or African American	1.662	0.921	3.002	0.092
Native Hawaiian or Pacific Islander	†	0.000		1.000
Unknown/not reported	0.756	0.464	1.231	0.260
Diabetes mellitus with non-insulin oral agents				0.234
Diabetes mellitus with insulin	2.105	0.818	5.417	0.123
No diabetes	1.087	0.583	2.028	0.792
Ventilator dependent	2.671	0.551	12.938	0.222
Congestive heart failure in 30 days before surgery	†	0.000		0.999
Hypertension requiring medication	1.303	0.870	1.952	0.198
Currently on dialysis (pre-op)	†	0.000		0.999
>10% loss body weight in last 6 months	2.804	0.889	8.844	0.078
Open wound/wound infection	2.610	1.019	6.682	0.046*
Systemic Sepsis	2.684	1.216	5.926	0.015*
Bleeding disorders	2.589	1.021	6.561	0.045*

Significant findings are in bold font.

* *P* value <0.05.

† Too high to be reported.

Cranioplasty complication rates did not vary significantly based on type (Table 5). Rates of overall complications (*P* < 0.001) and medical complications (*P* < 0.001) were significantly different between cranioplasties <5 cm and >5 cm (Table 6) (see table, Supplemental Digital Content 1, which lists the numerical value of specific complications by cranioplasty type, http://links.lww.com/PRSGO/B266) (see table, Supplemental Digital Content 2, which lists the specific complications by cranioplasty size, http://links.lww.com/PRSGO/B267).

**Table 5.** The Outcome of Overall, Medical, or Surgical Complications Was Compared between Alloplastic, Autologous, and “Other” Cranioplasty Groups

		Cranioplasty Type			
		Alloplastic (n = 543) (%)	Autologous (n = 57) (%)	Other (n = 99) (%)	Significance
Complication Type	Overall	28.00	24.60	27.30	0.86
	Surgical	3.50	3.50	2.00	0.75
	Medical	17.50	22.80	15.20	0.48

**Overall complication rates include medical complications, surgical complications, death, reoperation and readmission.

**Table 6.** The Outcome of Overall Complications, Medical Complications, Surgical Complications, and Death Was Compared between Procedures with Cranioplasty >5 cm and Those with Cranioplasty <5 cm

	Defect Size				
	>5 cm		<5 cm		
	n = 385*	%	n = 212*	%	
Overall complications†	85	22.10	79	37.30	**<0.001**‡
Medical complications	44	11.40	63	29.70	**<0.001**‡
Surgical complications	13	3.40	7	3.30	0.96
Death	8	2.10	7	3.30	0.36

Significant findings are in bold font.

*Two patients who had a >5 cm and <5 cm implant were excluded.

†Overall complications include death, reoperation, and readmission as well as surgical and medical complications.

‡*P* value < 0.001.

## DISCUSSION

In this analysis, 697 patients were identified in the NSQIP database as having undergone cranioplasty. The mortality within 30 days of the procedure was 3.0% and the presence of any complication 30 days after surgery was 27.4%. Comparable mortality and complication rates have been found in the literature.^6–8,12,13^

There are discrepancies in past reports on the complication profiles of different cranioplasty types. Past studies have demonstrated lower rates of surgical complications in synthetic implants as opposed to autografts,^14^ higher complication rates in non-autogenous versus autogenous grafts,^15^ or no difference between autografts and allografts.^16,17^ Our analysis found no significant differences in medical, surgical, and overall complications between the cranioplasty types.

While implant material did not appear to influence morbidity, implant size did. Larger implants were associated with higher overall complications (*P* < 0.001) and medical complications (*P* < 0.001). Other studies have also found a relationship between implant size and complications.^9,18^ Patients needing larger implants often have a higher degree of trauma and infection before cranioplasty surgery, which may contribute to their poorer postoperative outcomes.

Of the variety of variables evaluated in the multivariate analysis, age, bleeding disorders, open wound/wound infection, and systemic sepsis were found to significantly increase the risk of complication. Age has been found to increase postsurgical morbidity in cranioplasty and neurosurgical procedures as a whole.^13,19,20^ While some studies recommend that age alone should not be used rule out surgery,^20^ age should be included in the discussion with the prospective cranioplasty patient since for every 1 year increase in age the morbidity of cranioplasty increases by 1.4% (Table 4).

Open wounds (with or without infection) before surgery were also associated with an increased risk of complication. The odds of developing complication in patients with open wounds (relative to no wounds) were 2.61:1. Craniectomies and craniotomies, which are performed before cranioplasty, carry a sizeable risk of postoperative dehiscence.^21,22^ Wound dehiscence from these procedures impacts future wound healing, leads to hospital readmission, and increases risk of future infection.^21–24^ It follows that individuals with an open wound before surgery would have an increased risk of complications post-cranioplasty. While some would recommend delaying cranioplasty in the setting of open wounds and infection,^7^ this must be weighed with the risk of the neurological complications that can occur when cranioplasty is postponed.^25^

Bleeding disorders were also significantly associated with complications. Patients with bleeding disorders require complex management when undergoing surgical procedures, and the degree of risk surgery confers depends heavily on the severity of their disease and their medical regimen.^26^ This study found that the odds of patients with bleeding disorders developing complications when compared with controls were 2.59:1. As such, caution and appropriate intraoperative management are recommended for patients with bleeding disorders before cranioplasty.

Systemic sepsis—including sepsis, septic shock, and SIRS—was also associated with postoperative morbidity. The odds of developing any complication in septic patients versus non-septic patients were 2.68:1. Even outside of the surgical setting, septic patients are at drastically increased risk of death and acute organ failure.^27,28^ Subjecting such high-risk patients to surgery puts them at additional risk for death, lung, liver, and renal failure, as well as a host of other complications.^29^ Due to the high rates of complication following cranioplasty in septic patients, the patient and the surgical team should evaluate whether the procedure is worth the risks to the patient’s overall health.

### Limitations

This study is limited by the nature and scope of the NSQIP database. While this database contains comprehensive morbidity data 30 days postoperatively, outcomes beyond that point are lost. Past studies have followed patients from a couple of months to several years after cranioplasty to monitor for any complications.^3,14,17,30^ Many complications, such as bone resorption, infection, and exposure of implant material may take a longer time to manifest, and thus would be missed by this study.

Another limitation of this study is that a wide variety of implant materials are considered alloplastic implants—including titanium, polymethyl-methacrylate, polyether-ketone-ketone, and hydroxyapatite. However, there was no way to differentiate these further based on the NSQIP data. Different alloplastic materials may have different complication profiles.^12,31,32^ Thus, the heterogeneity of the alloplastic group should be taken into consideration in light of the similar complication profiles between the autologous, alloplastic, and “other” cranioplasty group.

Lastly, the univariate analysis results informed the multivariate model selection. More robust findings may have been made evident with a more sophisticated statistical model.

## CONCLUSIONS

In conclusion, cranioplasty is a morbid procedure, with a complication rate of 27.4% and a mortality rate of 3.0% in this national sample. Factors such as age, sepsis, open wound/wound infection, bleeding disorders, and size increase risk. Identification and modification of risk factors may guide operative timing and influence informed consent.

## ACKNOWLEDGEMENTS

The authors wish to thank Jennifer McGrath, MD, for her contributions throughout the project.

## Supplementary Material
