# Supplementary material for: Determinants of 30-day Morbidity in Adult Cranioplasty: An ACS-NSQIP Analysis of 697 Cases
Source: Plast Reconstr Surg Glob Open. 2019 Dec 11;7(12):e2562. doi: 10.1097/GOX.0000000000002562 (PMC7288897; doi:10.1097/GOX.0000000000002562)
Supplement: Supplementary file 1 [file gox-7-e2562-s001.pdf]

SDC1: This table lists the numerical value of specific complications by cranioplasty type.

Outcome	Alloplastic	Autologous	Other	Allo + Auto	Allo + Other	Total
**Surgical Complications**						
Occurrences Superficial Infection (SUPINFEC)	6	1	0	0	0	7
Occurences Deep Incisional SSI (WNDINF)	4	0	0	0	0	4
Occurrences Organ Space SSI (ORGSPSSI)	9	1	1	0	0	11
Occurrences Wound Disruption (DEHIS)	4	1	1	0	0	6
**Medical Complications**						
Occurrences Pneumonia (OUPNEUMO)	12	2	3	0	0	17
Occurrences Reintubation (REINTUB)	13	0	2	0	0	15
Occurrence Pulmonary Embolism (PULEMBOL)	4	0	0	0	0	4
Occurrence Failure to Wean From Respirator (FAILWEAN)	23	1	4	0	0	28
Occurrences Progressive Renal Insufficiency (RENAINSF)	4	0	0	0	0	4
Occurrences Acute Renal Failure (OPRENAFL)	2	0	0	0	0	2
Occurrences UTI (URNINFEC)	13	0	1	0	0	14
CVA/Stroke with neurological deficit (CNSCVA)	13	1	1	0	0	15
Occurrences Cardiac Arrest Requiring CPR (CDARREST)	3	0	0	0	0	3
Occurrences Myocardial Infarction (CDMI)	0	0	0	0	0	0
Occurrences Bleeding Transfusions (OTHBLEED)	45	7	4	1	1	58
Occurrences DVT/Thrombophlebitis (OTHDVT)	10	2	5	0	0	17
Occurrences Sepsis (OTHSYSEP)	12	2	3	0	0	17
Occurrences Septic Shock (OTHSESHOCK)	1	0	0	0	0	1
**Death, readmission, and return to OR**						
Death	14	1	6	0	0	21
Readmission	58	1	11	0	0	70
Return to OR	47	2	7	0	0	56
