# Supplementary material for: Determinants of 30-day Morbidity in Adult Cranioplasty: An ACS-NSQIP Analysis of 697 Cases
Source: Plast Reconstr Surg Glob Open. 2019 Dec 11;7(12):e2562. doi: 10.1097/GOX.0000000000002562 (PMC7288897; doi:10.1097/GOX.0000000000002562)
Supplement: Supplementary file 2 [file gox-7-e2562-s002.pdf]

SDC 2: This table list the specific complications by cranioplasty size.

**Outcome**	Cranioplasty size		
**Surgical Complication**	<5 cm	> 5 cm	Total
Occurrences Superficial Infection (SUPINFEC)	3	4	7
Occurrences Deep Incisional SSI (WNDINF)	2	2	4
Occurrences Organ Space SSI (ORGSPSSI)	7	2	9
Occurrences Wound Disruption (DEHIS)	1	4	5
**Medical Complication**			0
Occurrences Pneumonia (OUPNEUMO)	6	8	14
Occurrences Reintubation (REINTUB)	8	5	13
Occurrence Pulmonary Embolism (PULEMBOL)	1	3	4
Occurrence Failure to Wean From Respirator (FAILWEAN)	10	14	24
Occurrences Progressive Renal Insufficiency (RENAINSF)	3	1	4
Occurrences Acute Renal Failure (OPRENAFL)	1	1	2
Occurrences UTI (URNINFEC)	3	10	13
CVA/Stroke with neurological deficit (CNSCVA)	7	7	14
Occurrences Cardiac Arrest Requiring CPR (CDARREST)	2	1	3
Occurrences Myocardial Infarction (CDMI)	0	0	0
Occurrences Bleeding Transfusions (OTHBLEED)	22	32	54
Occurrences DVT/Thrombophlebitis (OTHDVT)	3	9	12
Occurrences Sepsis (OTHSYSEP)	6	8	14
Occurrences Septic Shock (OTHSESHOCK)	0	1	1
**Death, readmission, or Return to OR**			0
Death	8	7	15
Readmission	39	19	58
Return to OR	26	22	48
